# MaGelLAn 1.0: a software to facilitate quantitative and population genetic analysis of maternal inheritance by combination of molecular and pedigree information

**DOI:** 10.1186/s12711-016-0242-9

**Published:** 2016-09-10

**Authors:** Strahil Ristov, Vladimir Brajkovic, Vlatka Cubric-Curik, Ivan Michieli, Ino Curik

**Affiliations:** 1 Ruđer Bošković Institute, Bijenička cesta 54, 10000 Zagreb, Croatia; 2 Faculty of Agriculture, University of Zagreb, Svetošimunska cesta 25, 10000 Zagreb, Croatia

## Abstract

**Background:**

Identification of genes or even nucleotides that are responsible for quantitative and adaptive trait variation is a difficult task due to the complex interdependence between a large number of genetic and environmental factors. The polymorphism of the mitogenome is one of the factors that can contribute to quantitative trait variation. However, the effects of the mitogenome have not been comprehensively studied, since large numbers of mitogenome sequences and recorded phenotypes are required to reach the adequate power of analysis. Current research in our group focuses on acquiring the necessary mitochondria sequence information and analysing its influence on the phenotype of a quantitative trait. To facilitate these tasks we have produced software for processing pedigrees that is optimised for maternal lineage analysis.

**Results:**

We present MaGelLAn 1.0 (maternal genealogy lineage analyser), a suite of four Python scripts (modules) that is designed to facilitate the analysis of the impact of mitogenome polymorphism on quantitative trait variation by combining molecular and pedigree information. MaGelLAn 1.0 is primarily used to: (1) optimise the sampling strategy for molecular analyses; (2) identify and correct pedigree inconsistencies; and (3) identify maternal lineages and assign the corresponding mitogenome sequences to all individuals in the pedigree, this information being used as input to any of the standard software for quantitative genetic (association) analysis. In addition, MaGelLAn 1.0 allows computing the mitogenome (maternal) effective population sizes and probability of mitogenome (maternal) identity that are useful for conservation management of small populations.

**Conclusions:**

MaGelLAn is the first tool for pedigree analysis that focuses on quantitative genetic analyses of mitogenome data. It is conceived with the purpose to significantly reduce the effort in handling and preparing large pedigrees for processing the information linked to maternal lines. The software source code, along with the manual and the example files can be downloaded at http://lissp.irb.hr/software/magellan-1-0/ and https://github.com/sristov/magellan.

**Electronic supplementary material:**

The online version of this article (doi:10.1186/s12711-016-0242-9) contains supplementary material, which is available to authorized users.

## Background

The estimation of heritabilities, genetic correlations, effective population sizes, and other parameters that are central to quantitative and population genetics have, so far, exclusively depended on the existence of reliable genealogical information in pedigrees. The accurate and comprehensive acquisition of pedigree recordings, which is specific to livestock, laboratory, human, and some wild populations, was for a long time a factor that limited the feasibility and precision of quantitative and population genetic parameters estimates. Recent discovery of high-throughput genomic technologies, such as next-generation sequencing and microarrays, has made possible the sequencing of large regions (even genomes) or genotyping of thousands or millions of single nucleotide polymorphisms (SNPs) at a low cost. This has, in turn, enabled the analysis of the genetic architecture of complex traits without pedigree information. In humans [1, 2], laboratory [3, 4] and livestock [5, 6] populations, new technology developments are already used intensively, but are often still combined with pedigree information [7]. At the same time, the application of new technologies is continuously extending to a wider range of wild species [8–11].

To date, most of the research effort has been oriented towards the nuclear genome. With the exception of human populations, the impact of the mitogenome polymorphism (i.e. the polymorphism of the whole mitochondrial DNA sequence) on the phenotypic variability has been given almost negligible attention [12]. Although providing just a small part of the total DNA, the mitogenome is responsible for the regulation of a large portion of cellular adenosine triphosphate that is generated through oxidative phosphorylation, a metabolic pathway crucial for aerobic respiration [13]. Research on humans provided evidence that several mutations in the mitogenome lead to degenerative defects [13, 14], or have a strong influence on quantitative trait variations [15–18]. The main reason why the impact of the mitogenome on quantitative traits is understudied is that a very large number of sequences is required to reach the adequate power of statistical analysis. However, this is changing rapidly [19]. The most that has been done to infer the impact of the mitogenome on phenotypic variability concerns research in livestock [20–22] and humans [23] and for which pedigree maternal lineages were mainly modelled as random effects. These studies were based on the fact that, in mammals, transmission of the mitogenome is almost exclusively via the mother and that, in the absence of mutations, the same haplotype can be traced back to founders through maternal lineages. However, this approach is sensitive to pedigree errors, does not account for new mutations, and assumes that all maternal lineages are different. Furthermore, if present, the effects of single mutations cannot be distinguished. Improvements in sequencing efficiency and availability of mitogenome information at affordable prices offer new perspectives in the research related to the effects of the mitogenome on complex traits. For example, some commercial SNP chips that are routinely used for humans [18] and livestock [24] can also provide information on the mitogenome polymorphism. At the same time, sequencing of the whole mitogenome becomes feasible on a large scale [25, 26]. However, regardless of further molecular developments, the combined use of pedigree records and molecular information, from partial mitochondrial DNA (mtDNA) segments to the whole mitogenome, remains the optimal solution to evaluate the impact of the mitogenome polymorphism on quantitative traits.

Effective population size (N_e_) is a complex concept that can, concisely, be defined as the effective number of breeding individuals that explain the change in genetic variability (variance of allele frequency, inbreeding, heterozygosity loss or linkage disequilibrium) over time. Charlesworth [27] provides a more comprehensive and detailed discussion on various definitions of N_e_. Application of the N_e_ concept is important for the management and conservation of genetically small populations [28], for long-term prediction of selection responses [29] and for assessing the power of genome-wise association studies [30]. While N_e_ is mainly calculated for the autosomal genes, the concept can be extended to the sex chromosomes and mitochondrial inheritance [31, 32].

We present the MaGelLAn (maternal genealogy lineage analyser), which is a freely available software designed to help with testing of hypotheses that are related to maternal inheritance of quantitative traits by using pedigree and molecular mtDNA information. MaGelLAn is primarily intended for facilitating association analyses between polymorphisms of the mtDNA and quantitative traits, but also for facilitating the analysis of cytoplasmic models when mtDNA information is not available. Version 1.0 of MaGelLAn is a suite of four modules implemented as Python3 scripts. The principal functions of the modules are to: (a) analyze the concordance of the segregation of mtDNA haplotypes through the pedigree and detect haplotype discrepancies in the maternal line; (b) provide various statistics that are pertinent to maternal and haplotype lines (when the same haplotype is shared between multiple maternal lines) and to identify genealogical maternal lineages in order to impute mtDNA haplotypes to all members of a pedigree; (c) calculate effective population sizes of maternal lines and mtDNA haplotype lines in the reference population according to the method described in [33] (where, for maternal lines, effective population size is calculated based on pedigree information only, and for mtDNA haplotype lines, it is calculated based on molecular information only); and (d) suggest which individuals should be sampled to cover the maximum diversity within a pedigree and within a line, for which MaGelLAn provides an optimized sampling scheme that includes all animals that can be analyzed, with respect to their availability and the overall costs of molecular analysis.

With this combination of functionalities, MaGelLAn provides the imputation of mtDNA haplotypes to members of a pedigree with as much reliability as is possible within the available resources. This data can be further used as input to standard software for quantitative trait analysis.

## Implementation

MaGelLAn is a suite of four modules: *mag_verif*, *mag_stat*, *mag_calc* and *mag_sampl*. Modules are Python3 scripts that can be executed on any system that has Python3 interpreter installed. The modules are named according to their principal function, namely, verification of the pedigree correctness, viewing of the pedigree statistics with preparation for quantitative trait analysis, N_e_ calculation, and an aide for planning the sampling.

The scripts are user-friendly in the sense that all variables have self-explanatory names and the procedures are explicit and sequential with the result that the code is easy to read. If the need arises, the procedures in the scripts can be reused for different calculations that are based on lineage data, or any other application involving pedigrees. A reasonably competent Python programmer should have no trouble in modifying the scripts for that purpose, or to accommodate variations in input or output.

Prior to processing, all MaGelLAn modules check for the common errors in a pedigree. In case of an error, *mag_verif* module reports the error and prompts the user to correct it, while other modules abort the execution and direct the user to employ *mag_verif*.

### Software functionalities and an example session

Consider a case where we want to analyse population characteristics based on the pedigree data of the female lines. If mtDNA sequences are available at least for some individuals in a pedigree, then the following information can be obtained from the pedigree data of the female lines:

- verification of the pedigree correctness and measurement of the reliability of pedigree data;
- imputation of a haplotype to each individual in a pedigree, as a prerequisite for further analysis (mainly that of a connection between phenotype and mitochondrial haplotype);
- effective maternal population size;
- distribution of haplotypes over maternal lines and the corresponding effective haplotype population size.

The quality of the derived information depends on the number of available sequences relative to the total number of individuals in the pedigree/reference population. In the case of initial sequencing, or when the number of previously sequenced individuals is small, it would be useful to have a sampling plan that yields the highest haplotype diversity coverage for the given available resources (time and money).

There are two possible starting cases when dealing with a new pedigree regarding the available knowledge on mitochondrial sequences: the first is when there are no sequenced individuals at all, and the second is when mitochondrial sequences are already known for some individuals in a pedigree. Since our software is aimed mainly at the scientists with an interest in mitochondrial sequences in populations, it is reasonable to assume that the second case will be encountered more often in practice. Furthermore, the second case includes the first one as a special instance. Thus, we shall describe an example session under the assumption that haplotypes for some individuals in the reference population are already known.

**Fig. 1:**
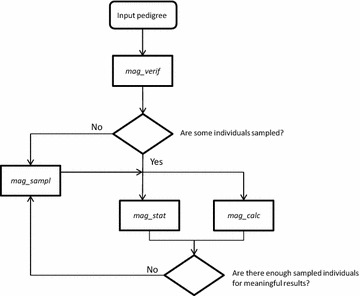
A typical workflow of the MaGelLAn modules with a new pedigree

A typical session with MaGelLAn software, when mitochondrial haplotypes for some individuals are known, is in Fig. 1. The input to our software is a standard pedigree file with a few modifications that are described in detail later in the text and in the accompanying manual, together with the specifics of the output files.

The first step should be to use the *mag_verif* module to check the pedigree consistency. If fatal errors exist in the pedigree, this module prompts the user to correct them. If some haplotypes are known, *mag_verif* checks for haplotype consistency in the maternal lines and calculates three numeric descriptors of pedigree data reliability. In the case of initial sequencing, *mag_verif* is used to prepare the pedigree file, or to verify its correctness, for input in the sampling stage.

Conceptually, there are two different and complementary final results of the MaGelLAn software, depending on the goal of the research. The first result is a thorough statistics of the distribution of individuals over maternal pedigree lines obtained with the *mag_stat* module. The main feature of this processing is the imputation of the haplotype to each individual in the pedigree. This information is essential, in particular, when investigating the correlation between haplotypes and phenotypes in a population. The second result is the calculation of effective population sizes, obtained with the *mag_calc* module, both for the maternal and the haplotype lines, in cases where multiple maternal lines share the same haplotype. The effective population sizes are important in research projects that deal with genetically small populations.

The quality of the results obtained with the *mag_stat* and *mag_calc* modules depends on the number of known haplotypes relative to the size of the reference population and the number of maternal lines. If the number of sequenced individuals is small then additional sampling is required, in which case an optimized sampling plan can be obtained with the *mag_sampl* module. In the case of initial sampling when there are no known haplotypes, the *mag_sampl* module should be used after the first verification step. The choice of the individuals for sampling is based on the assumptions that the number of samplings is defined by the available resources and that the coverage of the pedigree lines should be as diverse as possible within that number. We have implemented an algorithm that attempts to find an optimal coverage when there are no individuals with known sequences and, when some individuals are previously sampled, to find the individuals that would provide the best coverage when combined with the existing sampled individuals. In addition, we provide an option to restrict the samplings to individuals that are more accessible than the rest of the population.

Here, we present results regarding the speed of processing for different modules. All modules except the *mag_sampl* module perform only the operations that have a linear time complexity with respect to the input pedigree size. Thus, they are fast; on pedigrees of about 10,000 lines, the processing typically requires only a few seconds. The *mag_sampl* module includes a function for the selection of the best candidates, which requires a time length proportional to the sizes of each maternal line in the reference population to the power of three. For pedigrees of up to 10,000 lines, this amounts to a few minutes processing time. However, as pedigree size increases, the main factor that contributes to increasing processing time is the comparatively inefficient way that Python handles large data structures. As an example, we measured the times required for processing two cases: an average size rabbit pedigree with approximately 7000 lines [34], and the huge Croatian Holstein cattle pedigree with over 800,000 lines that was obtained from the Croatian Agricultural Agency (personal communication). The times needed for the processing of these two cases with *mag_calc* and *mag_sampl* modules on a 2.4 MHZ PC are 2 and 65 s for the rabbit, and 13 and 14 h for the Croatian Holstein cattle pedigree, respectively. The long processing times for the larger pedigree are still acceptable since pedigrees that comprise millions of individuals are fairly rare, while for the majority of cases the processing times are not an issue.

In the rest of this section, we provide details on the procedures used and the modules’ usage.

### Data input and output

**Fig. 2:**
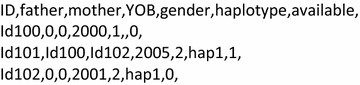
An example of valid first four lines in the input pedigree file. The *first line* is the header line. The position of the information in *columns* must match the position of the corresponding keyword in the header

All four MaGelLAn scripts require the same input pedigree file format. MaGelLAn modules accept the input *file name* as an argument in the command line. If the *file name* argument is absent, the program looks for the default input file “pdg_in.csv”. The input pedigree file must be in CSV (comma-separated values) format and the first line must be a header that includes five mandatory and two optional keywords. Mandatory keywords are [ID (individual), father, mother, YOB (year of birth), gender], and optional keywords are [haplotype, available]. The optional keyword “available” heads the column with information about the accessibility of an individual and its usage will be fully explained below in the subsection on the *mag_sampl* module. The place of keywords in the header line must match the column with the corresponding information in the pedigree file. An example of valid first lines in the input pedigree file is in Fig. 2. The pedigree may also include other data columns, and the order of the columns is irrelevant as long as the positions of the header keywords match their corresponding columns.

If a keyword is present but the corresponding column is missing, the program crashes. If a mandatory keyword is absent, the program also crashes. If the optional keyword “haplotype” is absent (together with the haplotype data), MaGelLAn modules remain operational but with a reduced functionality. The optional keyword “available” (with the corresponding data) is used exclusively in the *mag_sampl* module.

If an optional “reference_years.txt” file exists in the working directory, all MaGelLAn modules except *mag_verif*, accept it as an input file. This file stores, in two separate lines, the first and the last year of birth for the individuals included in the reference population. If the file is absent, the default (hard-coded in the scripts) years are used.

An optional “planned_number_of_sequencings.txt” file consists of a single number that denotes the intended number of samplings for the exclusive use in the *mag_sampl* module. If the file is absent, a hard-coded value is used.

Output files are textual files with self-explanatory names in the format “Output*Modulename*_*FileContent*.txt”. E.g., “OutputVerif_ConflictingIndividuals.txt” file is an output from *mag_verif* module that lists all individuals with conflicting haplotypes in the same maternal line. The details of the output content are presented in the following module descriptions and in the accompanying manual.

### Verification of the pedigree correctness: mag_verif module

The main functionality of this module is essentially the same as that of *mag_con_demo*, which is the initial tool for demonstration used in our previous work on computational pedigree analysis based on mtDNA [35]. Namely, *mag_verif* performs the verification of pedigree concordance with respect to mitochondrial haplotypes and maternal lines. Since all female descendants of a founder dam must have the same mtDNA haplotype, a conflict in haplotypes is an indication that there was an error in the pedigree maintenance (except for the very rare mutations). We had proposed three numerical descriptors as a means to measure the level of reliability of the pedigree data. These descriptors are three indices denoted with *HC* (for haplotype counting or conflicts), *IC* (for *informative* counting or conflicts) and *MISPLACED*. The verification procedure and the meaning of indices are fully explained in [35]. Here we shall briefly repeat the main ideas.

A *conflicting pair* is a pair of sequenced individuals that belong to the same maternal line, but have different haplotypes. A *conflicting individual* is the individual that has more pair wise conflicts than the individuals that are in conflict with it. *HC* index is defined as the percentage of non-concordant individuals among all haplotyped individuals.

An *informative individual* is an individual that is a member of a path between two haplotyped individuals in a pedigree tree. Only these individuals form the part of the pedigree that is included in the conflict analysis. *IC* index is defined as the percentage of non-concordant individuals among all informative individuals. This is useful in cases where the available samples do not cover all pedigree branches. Ideally, the individuals for sampling should be chosen in such a way as to maximise the number of informative individuals in a pedigree.

An individual is defined as *misplaced*, if it shows a conflict but has the same haplotype as its immediate haplotyped predecessor. Such a situation indicates that a whole pedigree branch is placed in the wrong line, and that, consequently, all conflicts in that branch are the result of a single error. *MISPLACED* index is defined as the percentage of misplaced individuals among all conflicting individuals. When summing up the number of conflicting individuals in a pedigree, if the number of the conflicts in a misplaced branch is reduced to 1, we obtain the *pruned* number of conflicting individuals.

The initial module *mag_con_demo* was written in C with custom-made data structures, which results in very fast processing but it lacks the flexibility and user friendliness of Python programs. In the present version, the output is modified in a manner that only the essential data is reported. It consists of three files “OutputVerif_Summary.txt”, “OutputVerif_ConflictingUnits.txt” and “OutputVerif_MisplacedBranches.txt”. Respectively, they contain: the summary of the data that are relevant for maternal lines analysis; the list of conflicting units with the corresponding number of conflicts (if conflicts exist in a pedigree); and the list of misplaced branches (if they exist in a pedigree). In the first version, *mag_con_demo* output also included the full list of conflicting pairs. If this is needed in any form, the Python script should be easy to modify.

Besides that of *mag_con_demo*, the *mag_verif* module includes the additional functionality to check for three standard types of errors that are usually found in pedigrees. Two types are fatal: cycles in a pedigree (an individual repeated as its own parent) and gender inconsistencies. When found, these errors are logged in the “ERROR_ALERT.TXT” file, one at a time, and the user is prompted to correct them and then the program execution stops.

The third type of error that MaGelLAn checks for is a case when an individual is listed as a parent, but does not have its own record in the pedigree. Such an error is non-fatal and it is automatically corrected in the following way. If an individual that does not have its own record is mentioned as a parent only once, it carries no information at all and is removed from the pedigree (the parent entry is set to “unknown”). If such an individual is mentioned twice or more, a new entry is created for it, since it carries information about relations for other individuals. This operation is performed silently, except that the lists of deleted individuals and newly created records are reported in the “autocorrection_log.txt” file. The described auto-correction feature is implemented in each MaGelLAn module.

### Extraction of maternal and mtDNA haplotype lines statistics: mag_stat module

The purpose of this module is to extract and present useful statistics for pedigree maternal lines. The extracted data are mainly the lists of pedigree individuals that are assigned to different maternal lines, with additional conditions that can be useful for the analysis of maternal lines. The complete lists of all individuals belonging to the same founder dam maternal line are stored in the file “OutputStat_DamLineMembership_1.txt”. This file, for each founder dam also displays the number of descendants and the haplotype. The file “OutputStat_DamLineMembership_2.txt” stores the same individual to founder dam relation in a format that is more amenable to processing in common spreadsheet programs. One particular application of such information is the possibility to impute a maternal haplotype to each individual in the pedigree, which further enables the evaluation of maternal (cytoplasmic) effects or even the effects of mitogenome sequence variability in quantitative genetic models.

The essential factors in the calculation of effective population size are the size of the reference population and the distribution of the founder dam lines within it. This distribution is exported in “OutputStat_DamLineMembershipAllInRefPop.txt” for all individuals in the reference population. Since only the female individuals are included in the calculation of the effective size of the maternal line, of equal interest is the distribution of the female individuals only in the reference population. This distribution is listed in the “OutputStat_DamLineMembershipFemaleOnlyInRefPop.txt” file.

Additional information that was proven useful in pedigree analysis is the quantitative breakdown of the founder dam lines representation in the reference population, which is exported in two files: “OutputStat_DamLinesWithFemalesInRefPop.txt” and “OutputStat_DamLinesWithOnlyMalesInRefPop.txt”. The founder dams that are represented in the reference population, but only with male descendants, are listed separately because such founder dams do not participate in the calculation of N_e_ for maternal lines, but do participate in its calculation for haplotype lines.

### Maternal and mtDNA effective population size: mag_calc module

The effective population sizes are calculated separately for maternal and mitogenome haplotype lines according to the method described in Alvarez et al. [33] using the genealogical (calculated only from the pedigree) or mtDNA molecular *probability of identity* (PI) [36] in founder and reference populations. Probability of identity of a female line, when there are *k* founder dams, is computed as PI = $\sum {q_{k}^{2} }$, where *q*_*k*_ is the frequency of the *k*th founder dam. In founder population *q*_*k*_ = 1*/k*, and in reference population *q*_*k*_ = (the number of female descendants of the *k*th dam in the reference population)/(all females in reference population). Probability of identity for haplotype lines is computed as PI = $\sum {q_{h}^{2} }$. In the founder population, *q*_*h*_ is the frequency of the *h*th haplotype among the founder dams. Each founder dam with unknown haplotype, as well as all her descendants, is assigned a unique haplotype. In the reference population, *q*_*h*_ = (all individuals in reference population with *h*th haplotype)/(all individuals in reference population). The haplotype for the individuals that are not sampled is imputed based on the sampled individuals in the same line. Therefore, the necessary requirement for calculation is that there are no conflicts in the pedigree.

Increase in identity is defined as:

$$\Delta PI = \frac{{PI_{r} - PI_{f} }}{{1 - PI_{f} }},$$

where *PI*_*r*_ and *PI*_*f*_ are probabilities of identity in the founder and reference populations, respectively. Effective sizes of female lines are finally computed as N_e_ = 1/*ΔPI*, both for maternal and mitogenome haplotype lines. The results, and all the data necessary for this calculation, as well as the basic pedigree statistics, are listed in the output file “OutputCalc_InputAndResults.txt”.

The symmetrical calculation of the effective size of paternal lines is included as well, although this is not in line with the focus on maternal lines of the MaGelLAn applications. If needed, the calculation of the effective size of the paternal Y-chromosome haplotype line should be easy to implement, too.

### Sampling strategy for molecular analysis: mag_sampl module

The reliability of the verification of the pedigree correctness in maternal lines, calculation of the N_e_ of the haplotype line, and imputation of a haplotype for the individuals in the maternal lines depend on the number and distribution of individuals that have been sampled and whose haplotypes have been determined. In the ideal case, all individuals in the reference population are sampled. In real life, the cost of sampling is an important factor, since the samples must first be collected in the field and then sequenced in the lab. Both of these actions involve considerable costs that restrict the amount of possible samplings. If the number of samplings is limited, it is essential that the distribution of the samples should cover a range of pedigree lines as wide as possible. We have developed and implemented an algorithm that, for a given intended number of sequencings, suggests a choice of individuals in the reference population that should be sampled, in such a way that it should provide the highest diversity coverage in the maternal lines of a pedigree.

The algorithm works in two stages; the first one calculates how many individuals should be sampled from each maternal line, and the second one chooses the particular individuals that form a group with the largest sum of mutual distances (within the line), thereby providing the best coverage of pedigree diversity.

Let *N* denote the planned number of sequencings or candidate slots, *L* the number of maternal lines in the reference population, *R* the size of the reference population (this is the pool of considered individuals), and *R*_*l*_ the number of individuals representing the *l*th maternal line in the reference population. The first goal is to find *N*_*l*_, the number of individuals that should be sampled for each line *l*.

If there are no previously sequenced individuals, the algorithm starts by assigning *N*_*l*_ = 1 for each line. In this way, all maternal lines are covered with at least one sample regardless of the size of *R*_*l*_. Then, the remaining (*N* − *L*) slots are distributed proportionally to the (*R*_*l*_ − 1) values for each line. In some cases, as with the initial sampling of a large population, a situation can arise where *L* > *N*. Then, one slot is assigned to the first *N* lines in the descending order of the size of *R*_*l*_. In this way, the *N* largest maternal lines are represented with one sample.

If some individuals in a pedigree are already sequenced and their haplotypes are known and included in the input pedigree file, the program attempts to distribute the intended number of sequencings proportionally over the sizes of *R*_*l*_ while taking the existing samples in account. Let *P*_*l*_ denote the number of previously sequenced individuals belonging to the *l*th maternal line. At the beginning, the algorithm assigns *N*_*l*_ = 1 for each line where *P*_*l*_ = 0. If there are *k* such lines, then the number of remaining slots (*N* − *k*) is added to the sum of all *P*_*l*_ and this new number is divided over the lines, such that it is as close as possible to the distribution proportional to the sizes of *R*_*l*_.

The second stage of the *mag_sampl* algorithm consists in picking out the actual *N*_*l*_ individuals for sampling that would provide the highest diversity coverage among *R*_*l*_ individuals in the reference population for each line *l*. This is achieved by choosing the set of individuals that are mutually the most distant in the pedigree tree, but that include a central node in the branch. The problem of finding a subset of tree nodes with the largest sum of distances is solved iteratively. The first step is to choose the first individual. The best representative of a line in the reference population should be the central individual in the corresponding pedigree tree branch. If we denote with *SD*_*l*,*i*_ the sum of the distances in the pedigree tree between individual *i* in line *l* and all other individuals in *l*, then the central individual is the one (or one of more) with the minimum *SD*_*l*,*i*_. The choice of the first individual is important when the intended number of samplings per line is small.

When the first individual is set, the remaining (*N*_*l*_ − 1) individuals are selected in (*N*_*l*_ − 1) iterations in such a way that the sum of the distances between them is the largest possible. The guaranteed optimal solution for this problem involves recursion and a dynamic programming paradigm. A code for this would be unreadable for the general audience. For this reason, we decided to use much simpler heuristics that reaches an optimal or nearly optimal solution in every case we have tested it for. At each iteration, the procedure chooses, in a greedy manner, the individual *j* with the largest *SD*_*P*,*j*_, where *P* is the set of already selected individuals. In plain words, we choose as the next individual in the set, the individual that has the largest sum of distances with the previously chosen individuals.

In the case when some individuals have known haplotypes, the previously sequenced *P*_*l*_ individuals are used as a seed group in the iterative procedure, thereby avoiding the need for selecting the first individual. The remaining (*N*_*l*_ − *P*_*l*_) individuals are then selected in the described iterative manner.

The individuals selected as the best candidates for sequencing are listed in the output file “OutputSampl_IndividualsForSampling.txt”, and the detailed information about maternal line sizes and computed values is given in “OutputSampl_DetailedInfo.txt”.

### Using the information about the available individuals

The harvesting of samples can be linked to different levels of difficulty for different parts of the population. Some individuals may be geographically more conveniently located then others or some locations (for example farms in livestock research) may provide samples at less cost than others. Alternatively, as was the case in our original study that motivated this approach, more samples have been harvested and are available, but only a restricted number of these can be sequenced. As a result, some individuals in the pedigree can be labelled as *available* and some as not available. We have included in our software the possibility to restrict the choice of individuals for sampling based on this availability criterion. The availability of an individual is denoted in an optional pedigree column headed with the *available* keyword. If an individual is available for sampling the value “1” is stored in the column. Anything else is a valid negation of availability, therefore the field can be left empty. If *mag_sampl* finds the keyword *available* in a header, the calculation of *N*_*l*_ is performed in the same way as described, but the choice of *N*_*l*_ or (*N*_*l*_ − *P*_*l*_) individuals is restricted to the available individuals. If this results in an overflow of the intended number of samplings over available individuals per line, *mag_sampl* does not attempt to redistribute the surplus slots but simply reports the details of the calculation and the numbers of remaining available individuals per line. Based on this information, the user can adjust the input number of sequencings or manually select the remaining individuals.

The restrictions on the selection imposed with the availability criterion are listed in the optional output file “OutputSampl_AvailabilityRestrictions.txt” that is created only if *available* data exists. If the *available* data column is not present, the available population is set to equal the reference population.

## Results and discussion

 Our motivation to develop the MaGelLAn software came from the lack of adequate tools for solving the problems that we encountered in our research on the influence of the mitogenome variability on quantitative traits in large populations. Initially, as the number of sequenced individuals from our pedigrees increased to hundreds, we needed a computational approach to the pedigree concordance problem. Consequently, we developed other components of MaGelLAn 1.0 to enable the preparation of the data that are required to analyse the impact of the mitogenome polymorphism on quantitative trait variation. In addition, MaGelLAn 1.0 calculates parameters, such as mitogenome and maternal lineage effective population sizes, which are useful in conservation management of small populations. Since quantitative trait analyses are based on large datasets, MaGelLAn 1.0 uses the appropriate computational methods that efficiently combine pedigree and molecular information. The main goal was to build the foundation of resources that can be further used to connect the mitogenome information with a large number of available phenotypes.

When dealing with nuclear markers and autosomal inheritance, several programs will have the same, or similar, functions as MaGelLAn 1.0. For example, programs exist that can correct pedigree errors [37] and calculate effective population sizes and co-ancestry coefficients [38, 39], but only for the pedigree data. Some other programs that are based only on molecular information, can calculate effective population size [40–42] and molecular coancestry [43], check for the consistency of pedigrees with molecular data [44, 45], or enable imputation of molecular information to pedigree-related individuals [46, 47]. However, complementary to all existing tools, MaGelLAn 1.0 is the only software intended to cover the specificities of the analysis of the mitogenome. In particular, it is aimed at the researchers who are interested in the analysis of the impact of the mitogenome on quantitative trait variability. We believe that MaGelLAn will help increase the understanding of this impact in populations with recorded pedigree information.

Regarding the code organization and software engineering aspects, MaGelLAn is implemented through user-friendly Python3 scripts with self-explanatory variable names and functions that are easy to understand and can be re-used in other similar applications. Some of the input and verification procedures that are used in all modules could have been implemented as external functions. However, some differences exist in the initial processing and we believe that this would decrease the readability of the code. In addition, our intention was to keep individual modules portable. With the intention to keep the software as easy to use and manage as possible, we chose to avoid the use of switches and the external routines.

An example of a typical use of the MaGelLAn suite when dealing with a new pedigree is presented in Fig. 1. However, depending on the state of the particular pedigree data, and the type of the information that is needed, the modules can be used in different combinations or separately. MaGelLAn 1.0. covers a comprehensive set of functionalities needed for the purposes of our research. We are open to the possibility that more functionalities will be added to MaGelLAn in time, and we invite other interested researchers to contribute with proposals of new functionalities or with their own code.

## Conclusions

MaGelLAn software has a combination of functionalities, some of which were not available previously, which are tailored to help researchers with pedigree analyses based on mitochondrial sequence data. The creation of the software was motivated by the specific needs we had in our lab for such research. The initial preparation of pedigrees is simple, i.e. it consists only of renaming the columns in the existing files, and, optionally, adding a new column with the availability of data. Once a pedigree is prepared, all MaGelLAn modules are easily applicable. We believe that MaGelLAn will be useful to researchers who deal with quantitative genetic analysis of maternal inheritance in populations with recorded genealogies i.e. for livestock, laboratory, zoo, some wildlife populations, and humans. The source codes of MaGelLAn 1.0., the manual, and pedigree example files are included as a supplement, and can be downloaded at [48], with a mirror at GitHub page [49]. The zip archive of the source codes only is accessible as additional material (Additional file 1).

## Availability and requirements

- Project name: MaGelLAn 1.0
- Project home page: http://lissp.irb.hr/software/magellan-1-0/
- Operating system(s): Platform independent
- Programming language: Python3
- Other requirements: None
- License: The software is free
- Any restrictions to use by non-academics: Written permission required

## Additional files

10.1186/s12711-016-0242-9 Zip archive of MaGelLAn1.0 source code consisting of four Python3 scripts.
